# The identification and characterization of the p.G91 deletion in CRYBA1 in a Chinese family with congenital cataracts

**DOI:** 10.1186/s12881-019-0882-z

**Published:** 2019-09-05

**Authors:** Dan Li, Qinghe Jing, Yongxiang Jiang

**Affiliations:** 1 Eye Institute, Eye & ENT Hospital of Fudan University, 83 Fenyang Road, Shanghai, 200031 China; 2 NHC Key Laboratory of Myopia (Fudan University), 83 Fenyang Road, Shanghai, 200031 China; 3 Key Laboratory of Myopia, Chinese Academy of Medical Sciences, 83 Fenyang Road, Shanghai, 200031 China; 4 Department of Ophthalmology, Eye & ENT Hospital of Fudan University, 83 Fenyang Road, Shanghai, 200031 China

## Abstract

**Background:**

Mutations in more than 52 genes have been identified in isolated congenital cataracts, the majority of which are located in crystalline and connexin (gap junction) genes. An in-frame one amino acid deletion in the beta-crystalline gene *CRYBA1* has been reported in several different Chinese, Caucasian and Iranian families of congenital cataracts. Further functional studies are needed to confirm the variant pathogenicity.

**Methods:**

The purpose of this study is to identify the genetic causes that contribute to congenital cataracts with esotropia and nystagmus in a Chinese family. Whole-exome sequencing was performed on samples from all five family members. The two brothers of the father and their daughters were then enrolled in the study, and 40 suspected variants were sequenced among the 9 subjects using Sanger sequencing. The mRNA and protein levels of *CRYBA1* in the lens epithelium from cataract patients and normal controls were compared using quantitative polymerase chain reaction (qPCR) and Western blot analyses. The wild-type and mutated forms (p.G91del) of *CRYBA1* cDNA were transfected into two types of cell lines, and the expression level of exogenous *CRYBA1* was measured by Western blot analysis. The exogenous CRYBA1 proteins were visualized by immunofluorescence staining.

**Results:**

In this two-generation family, all three descendants inherited congenital cataracts with esotropia and nystagmus from the father, while the mother’s lens was normal. After two rounds of sequencing, *CRYBA1* (c. 269–271 del, p.G91del) was identified as the mutation responsible for the autosomal dominant congenital cataract in the Chinese family. *CRYBA1* showed lower expression in cataract lenses than in control lenses. The deleted form (p.G91del) of CRYBA1 showed lower expression and was more aggregate to the cell membrane than the wild-type CRYBA1.

**Conclusions:**

We performed molecular experiments to confirm that the p.G91del mutation in *CRYBA1* results in abnormal expression and distribution of CRYBA1 protein, and this study could serve as an example of the pathogenicity of an in-frame small deletion in an inherited eye disorder.

**Electronic supplementary material:**

The online version of this article (10.1186/s12881-019-0882-z) contains supplementary material, which is available to authorized users.

## Background

Genetic counseling for various genetic diseases including congenital cataracts (CC) has begun in public hospitals and biotest companies in recent years. Commercially, approximately 150 genes have been compiled into one target-capture chip, the so-called “abnormal lens diagnosis chip”, including genes from isolated and syndromic cataracts, ectopia lentis, and microphthalmia. Black et al. reported that 115 CC-related/suspected genes were captured and sequenced for CC prediction, and the overall pick-up rate was 75% [1]. Due to the rapid development of sequencing technology, the cost of whole-exome sequencing (WES) has dropped to approximately 600 US dollars and has become a mainstream method for Trio testing. The advantage of WES is that it covers the coding regions of the whole genome, not only the “known” hundred or so. Compared with the results from targeted sequencing, data from WES provide many more candidate genes, although only the known genes are analyzed as a first priority. When the primary scan is negative, commercial services normally end at this step and report a negative result. However, for the purpose of genetic research, investigators need to expand the analysis pool empirically, by exploring the extensive “unknown” genes to find the novel gene that relates to the hereditary disease.

In the current study, we aimed to identify the genetic factors of CC with esotropia and nystagmus in a Chinese family. First, we performed WES on samples from five members of a two-generation family with a direct relationship: father, mother and three children, with the father and three children affected with CC, esotropia and nystagmus. After the primary scan, the in-frame deletion of 3 base pairs (bp) (c. 269–271 del, p.G91del) of *CRYBA1* was identified as the disease-related variant. *CRYBA1* encodes two proteins: βA1- and βA3-crystallins, belonging to β-cryallins, which are the most abundant water-soluble crystallins in the lens [2]. This 3 bp deletion in the exon 4 would cause the deletion of glycine at the 91st amino acid. Although in-frame small insertions/deletions (indels) are harmless in the majority of cases, this indel has been found in eight other reports of autosomal dominant CC in the last two decades (summarized in Table 1) [3–10]; five of these cases were Chinese patients, and the others were Swiss, English and Iranian patients. As shown in Table 1, in these studies, the main genotyping methods were short tandem repeat (STR) marker scan or direct sequencing of cataract candidate genes, and only Reddy et al. performed biochemical experiments to assess the structural effect of this indel [5], indicating that this recurrent 3 bp deletion in *CRYBA1* might cause malfunction of the protein.

**Table 1:** Summary of the previously reported CRYBA1 △G91 mutation in autosomal dominant congenital cataracts

	Phenotype	Race	Genotyping method	Functional study	Reported year	Ref.
1	Nuclear cataract	Swiss	STR of cataract genes and regions		2004	[3]
2	Nuclear cataract	Chinese	STR of 12 candidate gene		2004	[4]
3	Lamellar cataract	English	Genome wide STR	CRYBA1 mutant protein solubility analysis; circular dichroism spectroscopy	2004	[5]
4	Pulverulent cataract	Chinese	Genome wide SNP		2007	[6]
5	Nuclear catarct	Chinese	STR of 26 candidate genes		2011	[7]
6	Nuclear cataract	Chinese	12 candidate gene sequencing		2011	[8]
7	Nuclear cataract	Iranian	4 candidate gene sequencing		2016	[9]
8	Nuclear cataract	Chinese	One gene sequencing		2018	[10]
9	Nuclear cataract/ esotropia/ nystagmus	Chinese	Whole exome sequencing	CRYBA1 mutant protein expression and distribution	2019	^a^

^a^The current study

## Methods

### Patients

**Fig. 1:**
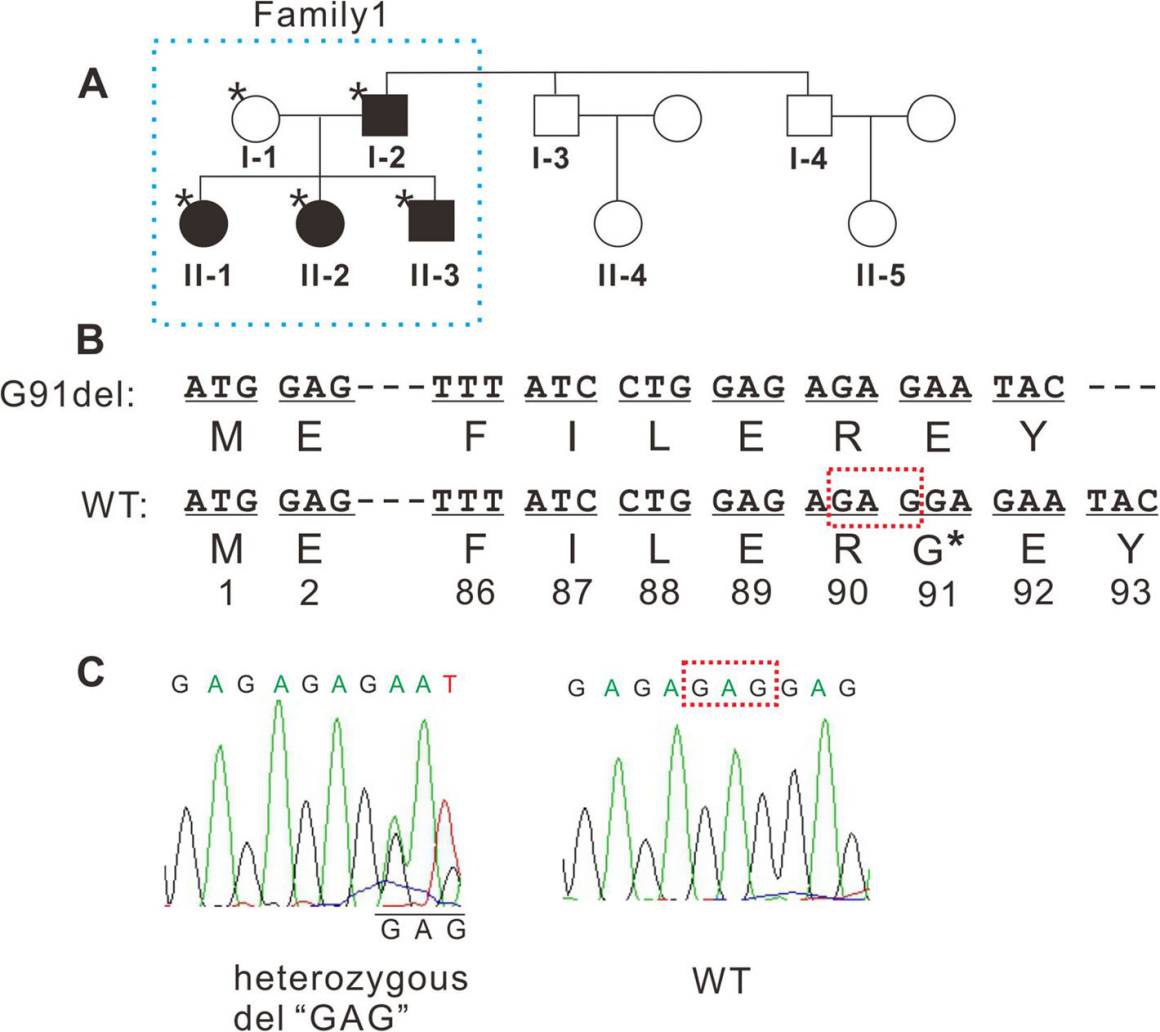
Overview of CRYBA1 mutation in the family. (**a**) Pedigree of the two-generation family. Filled symbols indicate individuals affected with CC. In individuals marked with an asterisk, the genotype was determined by exome sequencing. (**b**) Diagram of the genetic and amino acid sequences of WT and deleted mutant CRYBA1. The “GAG” box is the deleted nucleotide sequence. (**c**) Results of Sanger sequencing of CRYBA1 from affected and unaffected individuals are presented by chromatogram images

**Fig. 2:**
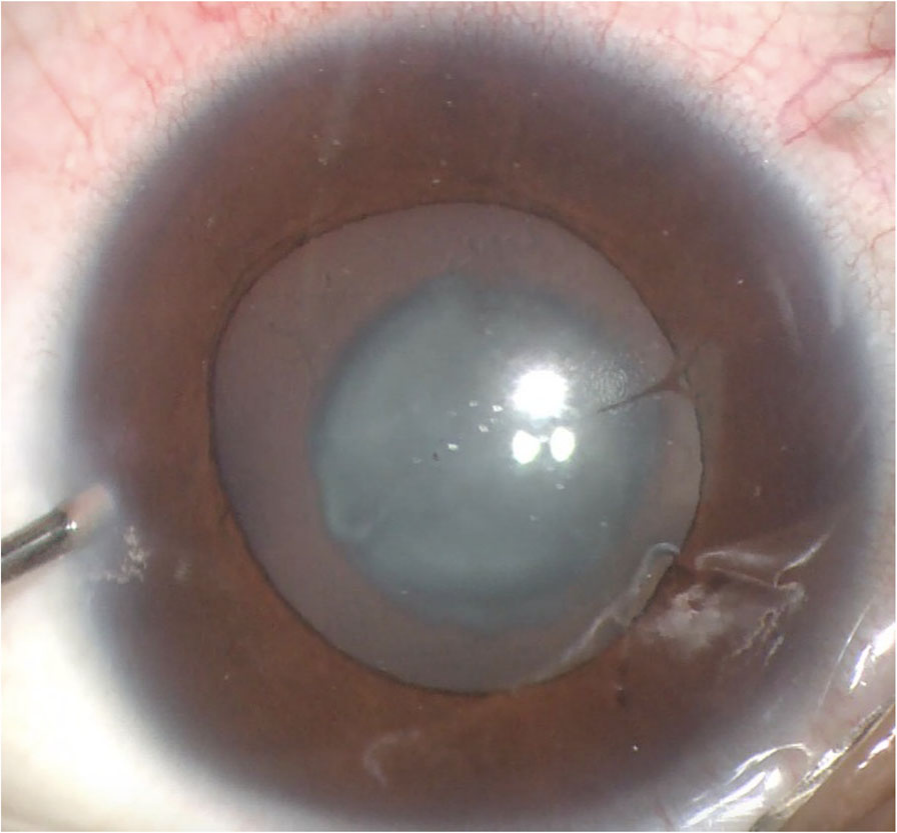
Ocular photograph of the proband (II-1). The image was captured from the surgery video, which revealed the opacity of nuclear cataracts

**Fig. 3:**
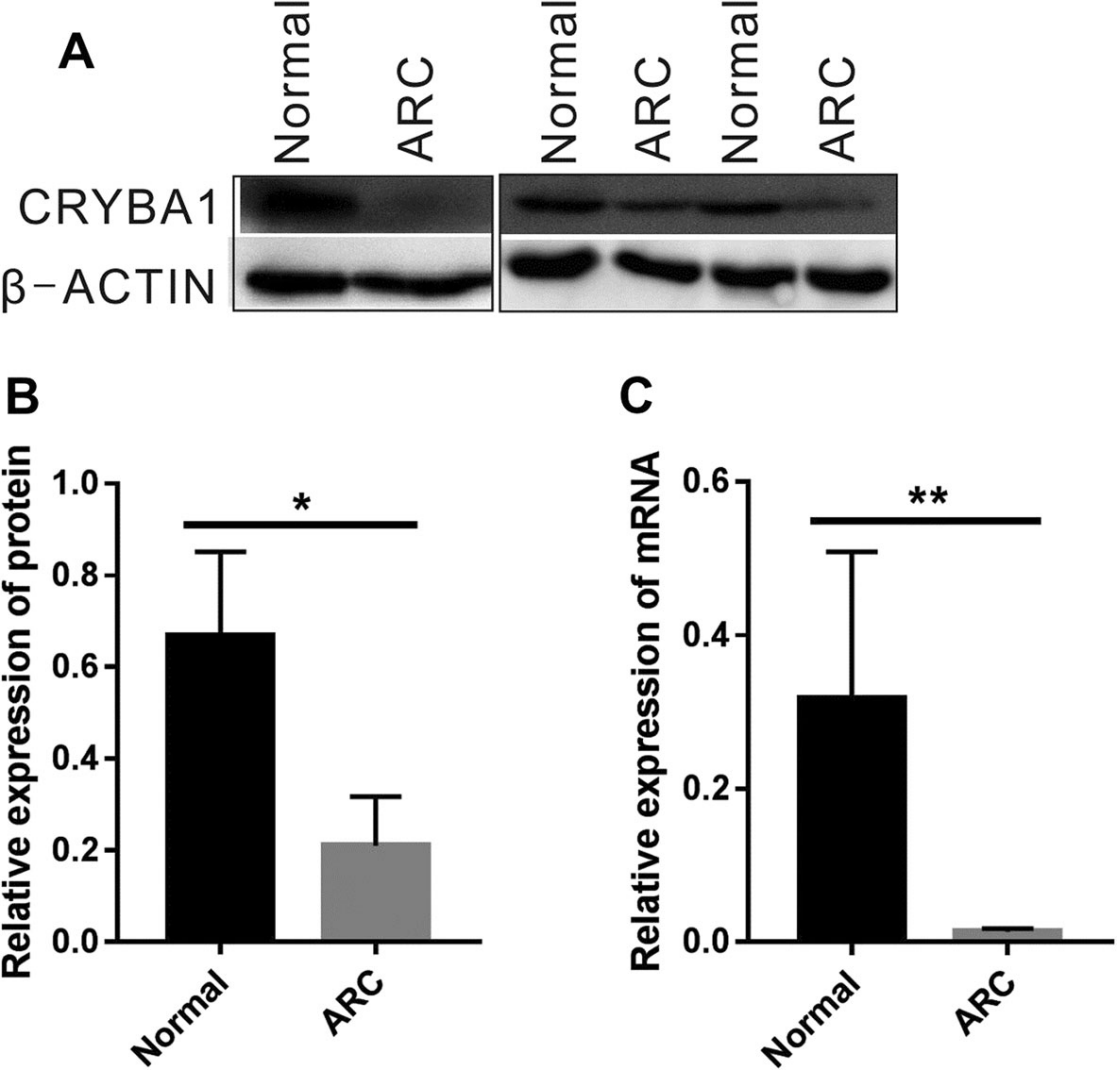
CRYBA1 is expressed less in cataract lens epithelium. (**a**) Western blot images of CRYBA1 in ARC and normal control lens epithelium. Three samples from each group are presented. β-Actin was used as an internal control. (**b**) Quantification of the gel images of the Western blots in (**a**) (*n* = 3). (**c**) The mRNA level of CRYBA1 was measured by qPCR in ARC and normal control lens epithelium. β-Actin was used as an internal control (n = 3). Student’s *t* test, *P < 0.05, **P < 0.01

**Fig. 4:**
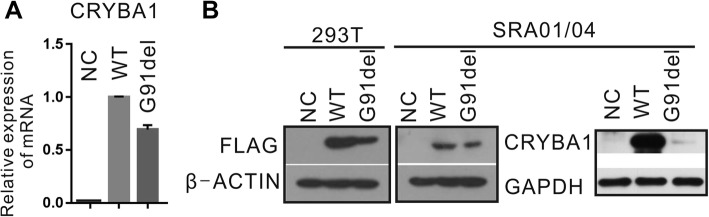
The CRYBA1 p.G91del mutation reduced its expression in two cell lines. (**a**) After the WT and deleted forms of CRYBA1 cDNA constructs were transfected into SRA cell lines, the relative mRNA level of CRYBA1 was quantified by qPCR. β-Actin was used as internal control. The WT group was used as sample control (n = 3). (**b**) The protein levels of CRYBA1 were measured by Western blot. In 293 T and SRA cells, the exogenous CRYBA1 was detected using anti-FLAG antibody; In SRA cells, the general CRYBA1 protein level was also measured using anti-CRYBA1 antibody. β-Actin or GAPDH were used as internal control. NC, negative control: transfection reagent only

The two-generation family includes eleven family members, nine of whom (Fig. 1a, I-1-4, II-1-5) participated in the study. Four family members were diagnosed with CCs with esotropia and nystagmus and underwent thorough ophthalmological examinations, including slit-lamp examination, fundus photochromy, optical coherence tomography (OCT) and visual evoked potential (VEP). Genomic DNA was extracted from blood samples from all nine participants. In Fig. 1a, genotype information with an asterisk indicates the individual whose sample was used for WES analysis.

Eighteen age-related cataract (ARC) patients (8 male and 10 female, aged 64.1 ± 4.5 years) agreed to donate the anterior capsule pieces from their cataract surgery to the study. The normal control capsular samples from 18 donors (12 male and 6 female, aged 60.2 ± 5.2 years) were provided by the eye bank of the Eye and ENT Hospital of Fudan University. The capsular piece of the normal donor lens was collected from the anterior part, the same site at which the capsule samples collected in the cataract surgery, as previously described [11].

### Sequencing analysis

For WES, libraries for sequencing on a HiSeq2000 instrument (Illumina Inc., USA) were prepared from genomic DNA and exome sequences enriched with SureSelect Human All Exon 50 M (Agilent Technologies, USA) according to the manufacturers’ instructions. The bioinformatic analysis of the raw data (paired-end reads) has been described previously [12]. In addition, the variants with the allele frequency > 1% in gnomAD database were excluded (https://gnomad.broadinstitute.org/). After a series of filtering steps, 40 sites in 38 genes (Additional file 1: Table S1) were selected for further analysis. Both strands of the selected regions were sequenced by Sanger sequencing following polymerase chain reaction (PCR) amplification on an ABI 3730 Genetic Analyzer (Applied Biosystems Inc., USA). The results of Sanger sequencing were analyzed and presented using Chromas software (https://technelysium.com.au/wp/chromas/).

### Cell culture and transfection

The human lens epithelial cell (LEC) line SRA01/04 (abbreviated as SRA in this study) was authenticated using short tandem repeat (STR) profiling, and the data of the STR analysis are provided in Additional file 2 (Shanghai Biowing Applied Biotechnology, China). SRA cells and embryonic kidney cell line 293 T cells were cultured in DMEM (#11995065, Gibco, USA) supplemented with 10% fetal bovine serum (#10099141, Gibco) under humidified air containing 5% CO_2_ at 37 °C. To overexpress the *CRYBA1* constructs in these two cell lines, cDNA sequences of the human *CRYBA1* (RefSeq NM_005208) wild-type and G91del mutant with the FLAG sequence “GATTACAAGGACGACGATGACAAG” at the N-terminus were cloned into the eukaryotic cell expression vector pcDNA3.1 (Invitrogen, USA). Cells were transfected using the transfection reagent (#C10511, RiboBio, China) according to the manufacturer’s instructions. In each well in a 6-well culture plate, 2.5 μg of plasmids were included in the transfection mixture.

### Quantitative PCR (qPCR)

Capsular pieces from three subjects were pooled into one sample. Total RNA from the patients’ lens epithelium or cultured cells was extracted using an RNA extraction kit (#CW0581, CoWin Biosciences, China) and reverse transcribed into cDNA using a cDNA synthesis kit (#CW2582, CoWin Biosciences) according to the manufacturer’s protocols. mRNA levels of the selected genes were quantified by SYBR Green-based quantitative PCR (qPCR) kit (#CW2601, CoWin Biosciences) on an ABI 7500 analyzer (Thermo Fisher Scientific). The relative mRNA expression between the target genes and the internal β-actin control was calculated using the comparative cycle threshold (CT) method (2^-△CT^). The relative mRNA level between samples was calculated using 2^-△△CT^ method.

### Western blot

Capsular pieces from three subjects were pooled into one sample. Western blot analysis was performed according to standard methods as previously described [13]. The primary antibodies included CRYBA1 (1:1000, #NBP1–33010, Novus Biologicals, USA), FLAG (1:1000, #8146, CST, USA) and CRYBA4 (1:1000, NBP1–32741, Novus). β-Actin (#A3854, Sigma-Aldrich, USA) or GAPDH (#5174, CST) served as the internal control. Secondary antibodies were goat anti-mouse or goat anti-rabbit IgG (H + L) poly-horseradish peroxidase secondary antibody (Jackson Immunoresearch, USA). The dilution of the secondary antibodies was 1:10000.

### Immunofluorescence staining

Cells used for immunofluorescence staining were seeded after a glass cover (#48380–080, VWR Scientific, USA) was placed into the bottom of the culture dish. Forty-eight hours after cell transfection, the glass covers were washed with phosphate buffer solution (PBS) and fixed with 4% paraformaldehyde for 15 min. Cells attached to the glass covers were then permeabilized with PBS containing 0.3% Triton X-100 for 15 min, followed by incubation in PBS containing 5% goat serum and 0.1% bovine serum albumin for 1 h to block nonspecific protein binding. Then, the cells were incubated with the FLAG primary antibody (1:100, #8146, CST), CRYBA1 (1:100, #NBP1–33010, Novus Biologicals) at 4 °C overnight. The next day, after washing with PBS, the cells were incubated with Alexa Fluor 488-conjugated goat anti-mouse IgG secondary antibody (1:1000, #R37116, Thermo Fisher, USA) for 1 h at room temperature. Hoechst 33258 (1:2000, #H3569, Invitrogen, USA) was used to visualize the nucleus. Finally, the glass cover was removed from the bottom of the culture dish and placed onto a glass slide with the cells facing downward. The cells were then observed using the Cell Observer microscope (Zeiss, Germany), and the images were captured using ZEN 2012 software (blue edition).

## Results

### Clinical features

We identified a two-generation Chinese family with a diagnosis of bilateral CC with esotropia and nystagmus (Table 1). Fundus photochromy, OCT, and VEP were performed on CC patients, the results showed no other ocular or systemic abnormalities. The pedigree of the family suggests a de novo mutation in subject I-2, with an autosomal dominant inheritance pattern in Family 1 (Fig. 1a). The nuclear cataract phenotype is presented in Fig. 2, the image of which was captured from the cataract surgery video of subject II-1.

### Identification of the genetic mutations

WES analysis of all five members in Family 1 followed by a series of bioinformatic analyses resulted in the identification of the *CRYBA1* mutation (c. 269–271 del, p.G91del). The reasons for the identification of this mutation include the following: 1) *CRYBA1* is a known cataract gene, 2) this deletion has been reported in multiple families and 3) this deletion is not present in ExAC or the 1000 Genome Project, although according to the normal criteria, it would be functionally moderate as it does not cause a frameshift or change the subsequent amino acid sequences. In addition, 40 variants in 38genes co-segregated with the CC phenotype are predicted to impair the gene function, including point mutations that would cause amino acid changes or gain stop codons and insertions or deletions that would cause a frameshift or affect exon splicing (Additional file 1: Table S1). Although the functions of most of these genes are unclear and can rarely be related to lens diseases, *CTBP2* is possibly linked to cataracts based on a previous report indicating a critical role of this gene in the endothelial to mesenchymal transition (EMT) in LECs [14]; *CRYBG2* (also known as *AIM1L*), encoding gamma crystallin domain-containing protein 2, might be related to the lens structure, but no phenotypic variant has been reported for this gene.

We then enrolled four more subjects from the other two subfamilies, including two brothers of I-2 in Family 1 (I-3 and I-4) and their daughters (II-4 and II-5), and we analyzed the suspected genetic sites using the Sanger sequencing method. The results of Sanger sequencing indicated that only the *CRYBA1* p.91del mutation was confirmed to co-segregate with the phenotype among all the participants, while each of the variants listed in Additional file 1: Table S1 has been detected in the unaffected subjects. Then we used MutationTaster to analyzed the functional effect of this deletion [15], and found that the p.G91del in *CRYBA1* is predicted to be “disease causing”.

### CRYBA1 expression is downregulated in cataract lenses

To investigate the expression levels of *CRYBA1* in cataract patients and normal controls, we collected capsule pieces from the cataract surgeries and the eye bank and used them for quantification studies. Ideally, the capsular samples from CC patients should be compared to those of age-matched (usually under five years on average from the database of our hospital) controls. However, it is very difficult to obtain lens samples of normal controls at such a young age. For this reason, we used capsular lens samples from ARC and normal subjects instead. In Fig. 3a and b, the protein levels of CRYBA1 in three groups of ARC and control samples are presented as the Western blot bands and quantitative columns; Fig. 3c shows the mRNA level of *CRYBA1* as measured by qPCR assay. These results indicate that the expression level of *CRYBA1* is significantly lower in the lens of ARC patients than in normal subjects.

### The p.G91del mutation of CRYBA1 reduced expression at the protein level

**Fig. 5:**
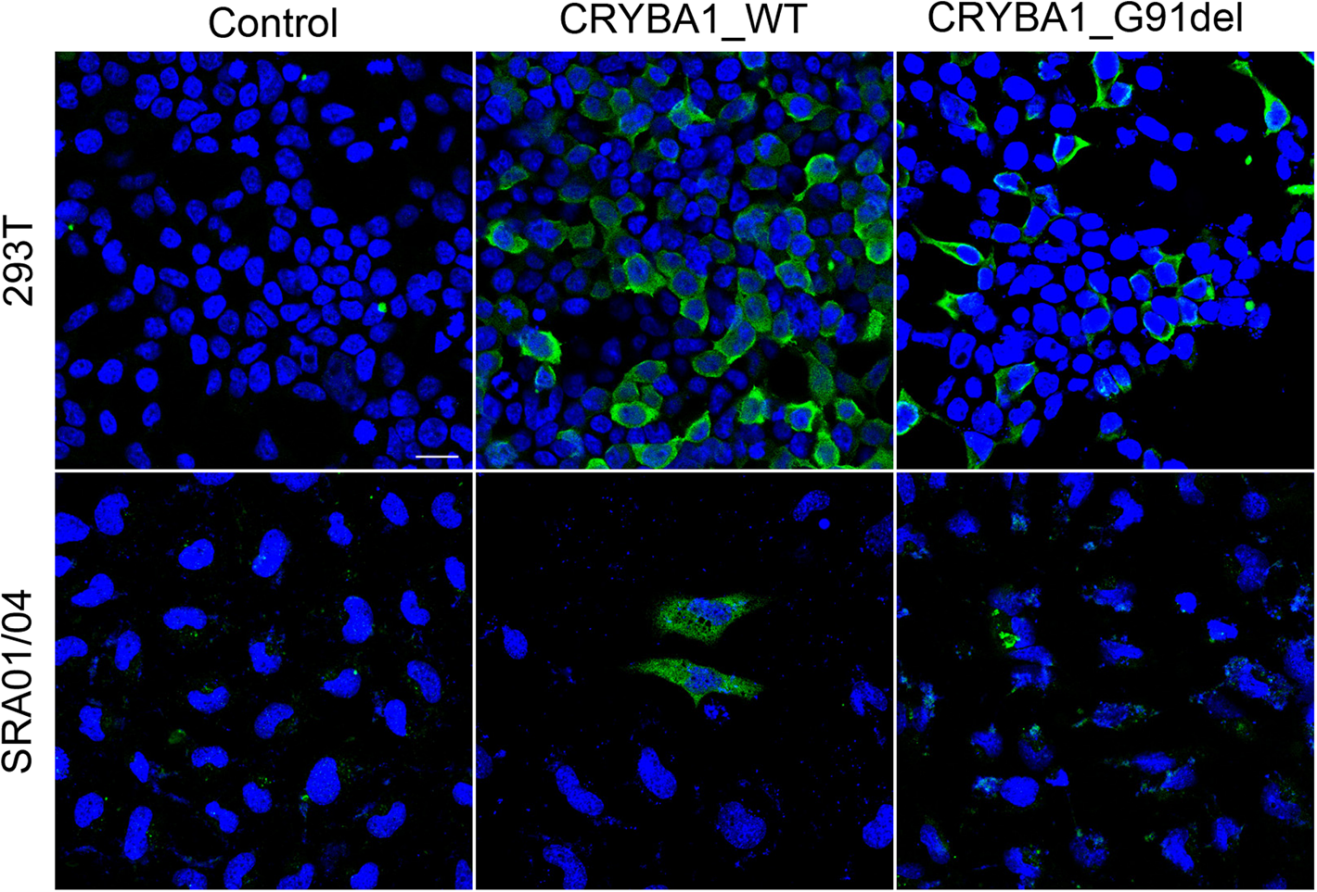
Immunofluorescence staining of exogenous WT and mutated CRYBA1 in 2 cell lines. After the WT and deleted forms of CRYBA1 cDNA constructs were transfected into 293 T and SRA lines, cells were stained with FLAG primary antibody. p.G91del mutation led to greater aggregation of the protein at the cell membrane. Blue: nuclear; green: exogenous CRYBA1, scale bar: 20 μM

To investigate the functional effect of p.G91del of *CRYBA1*, we conducted transfection of plasmid constructs into two types of cell lines. The deletional mutant and wild-type (WT) cDNA sequence of *CRYBA1* were cloned into the eukaryotic expression vector with a FLAG tag at the N-terminus. Both plasmids were transfected into two cell lines: SRA and 293 T cells. In the WT and G91del mutant *CRYBA1* plasmid transfected cells, both plasmids increased *CRYBA1* mRNA to more than 10^6 times (△△CT > 20), and the mRNA level between WT and mutant is comparable (Fig. 4a), while the protein level of mutant CRYBA1 was dramatically decreased (Fig. 4b). For this reason, we believe that the dysfunction of mutant CRYBA1 is mainly at the protein level. In Fig. 5, the mutant CRYBA1 proteins are not only expressed less but are also more aggregated to the plasma membrane, while the wild-type CRYBA1 proteins are distributed more evenly in the plasma.

In addition, considering the genetic compensation in response to gene mutations, we checked the closely related crystallin CRYBA4 in the CRYBA1-overexpressed SRA cells. Interestingly, the protein level of CRYBA4 in the WT CRYBA1 group is lower than that in mutant CRYBA1 group and the non-transfected control (Additional file 3: Figure S1), suggesting that the overload of CRYBA1 would reduce the production of CRYBA4, while the mutant type of CRYBA1 was not able to induce such reduction. However, we are not able to check the CRYBA4 level in the lens of the patients in this CRYBA1-mutated family due to the shortage of their lens capsule samples.

## Discussion

According to the American College of Medical Genetics and Genomics (ACMG), the Association for Molecular Pathology (AMP) and the College of American Pathologists (CAP), sequence variants should be interpreted according to specific rules and consequently categorized into one of six groups, as described in supplementary Additional file 4: Figure S2 (this figure was simplified from a reference guideline [16]).

In recent years, WES has been used as a mainstream method in genetic screening of inherited diseases; it has identified dozens of novel mutations in congenital cataracts, and the number is continuously increasing. In this study, we used WES to identify mutations among the five members of a two-generation Chinses family with CC, esotropia and nystagmus and found that the *CRYBA1* mutation (c. 269–271 del, p.G91del) is the top candidate on the list of potential causative mutations. However, this in-frame one amino acid indel is categorized as a non-truncating variant, whose pathogenicity is often more difficult to predict, as it does not cause a frameshift or change the following amino acid sequence [17]. For this reason, we suspected other possible causative mutations based on their co-segregation with the disease and the damage score predicted by the functional prediction tools. This finding raises a more general question: in the identification process of causative mutations, is it necessary to assess other candidate variants when a mutation in the “known gene” is detected? For example, *CTBP2* is probably related to the lens pathology, and the c.1879 del would cause a frameshift, but this mutation would normally be ignored when a crystallin gene mutation is detected in CC. To assess the variants from the “unknown genes” in this case, we included more family members as illustrated in Fig. 1a, and found that only the *CRYBA1* indel completely co-segregates with the phenotype in all the participants. After these tests, we concluded that this is very likely a disease-causing mutation. Specifically in this case, the CC patients also present esotropia and nystagmus, which raised the possibility that this indel may also be responsible for the malfunction of ocular muscular movement. However, in the studies on genetic screening of esotropia and/or nystagmus, there is only one report finding the *CRYBA1* c.594G > A:p.(Trp198Ter) mutation in families with nystagmus [18]. In addition, other families with the p.G91del mutation did not show such phenotype (summarized in Table 1). Therefore, we believe this deletion may not be the causing mutation responsible for esotropia and nystagmus.

Besides the p.G91del mutation we reported here, together with 8 other reports [3–10], the other main group of *CRYBA1* mutations have been identified in congenital cataracts: the mutations in the first two bases at the donor splice site of intron 3 (IVS3 + 1 G > A, IVS3 + 1 G > T, IVS3 + 1 G > C and IVS3 + 2 T > G) [8, 19–25]. Unlike the p.G91del mutation as summarized in Table 1, the subtypes of cataract caused by splice mutations are more variable, including nuclear [21], suture [20, 22], posterior polar [23] and progressive nuclear and cortical [25]. Of note, even the affected members with the same “IVS3 + 1 G > A” mutation in one family pedigree would present different cataract phenotypes [24]. In addition, a 2-bp deletion (c.590-591delAG) in exon 6 of *CRYBA1* was identified in five members with nuclear cataract in a Chinese family [26].

Although the *CRYBA1* mutation p.G91del has been linked to several families worldwide, the molecular function of this mutation has not been confirmed, although Reddy et al. performed structural studies and found a decreased solubility of the mutant protein [5], and Sergouniotis et al. used “integrative protein structure modeling” to determine that this single residue deletion (Gly91) is in an edge strand in β-sheets and is therefore likely to destabilize the protein [27]. Moreover, Sergouniotis et al. found that a small in-frame indel is quite common in inherited eye disorders, including cataract and retinal dystrophy [27]. Our interest in this mutant is in determining whether this in-frame deletion would affect cellular functions and cause the disease. Through overexpression of the wild-type and deleted forms of *CRYBA1* cDNA plasmids, we found that the protein level of CRYBA1 was evidently decreased by the p.G91del mutation, as presented in Figs. 4 and 5, suggesting that this one amino acid deletion could decrease the protein stability and might consequently lead to lens structural protein misfolding and denaturation. Besides, we observed that the mutated CRYBA1 protein would be more aggregate to the submembrane compartment (Fig. 5), most possibly results from 1) the significant decrease in its level in the cell [28] and 2) the structural misfolding of the protein [29]. However, manifestation of the cataract phenotype in a genetically modified animal model, most frequently the mouse model, is needed to provide confirmative evidence of this single residue deletion being the causative mutation of the disease.

## Conclusion

In the present study, we added one more report of CC linked to the p.G91del of *CRYBA1* in a Chinese family with esotropia and nystagmus, investigated the effect of this in-frame deletion mutation in vitro and concluded that it would affect the production and distribution of this lens structural protein.

## Additional files

Additional file 1: **Table S1.** 40 variants have been predicted to be pathogenic. (DOCX 18 kb)

Additional file 2: Cell Line Authentication Report STR Profiling. (PDF 221 kb)

Additional file 3: **Figure S1.** Overexpression of CRYBA1 would reduce CRYBA4 production. After the WT and deleted forms of CRYBA1 cDNA constructs were transfected into SRA cell lines, the protein levels of CRYBA4 were measured by Western blot. GAPDH was used as internal control. NC, negative control: transfection reagent only. (JPG 42 kb)

Additional file 4: **Figure S2.** Six categories with increasing severity of pathogenicity. Re-edited from Fig. 1 in [16]. (JPG 226 kb)

## Data Availability

The WES data is available from the corresponding author on reasonable request following approval of the ethics committee of Eye & ENT Hospital of Fudan University.

## Abbreviations

ARC: Age-related cataract
CC: Congenital cataract
indel: Insert/deletion
LEC: Lens epithelial cell
OCT: Coherence tomography
STR: Short tandem repeat
VEP: Visual evoked potential
WES: Whole-exome sequencing
