# Supplementary material for: The identification and characterization of the p.G91 deletion in CRYBA1 in a Chinese family with congenital cataracts
Source: BMC Med Genet. 2019 Sep 5;20:153. doi: 10.1186/s12881-019-0882-z (PMC6727356; doi:10.1186/s12881-019-0882-z)
Supplement: Supplementary file 1 — Table S1. 40 variants have been predicted to be pathogenic. (DOCX 18 kb) [file 12881_2019_882_MOESM1_ESM.docx]

Table S1. 40 variants have been predicted to be pathogenic.

|  | **Gene** | **Chr** | **NM** | **Mut_pos** | **Pro_info** | **Rs** |
| --- | --- | --- | --- | --- | --- | --- |
| 1 | CRYBG2 | chr1:26671545-26671578 | NM_001039775 | c.1571(exon2)_c.1604(exon2)del | p.L524Sfs*15 | na |
| 2 | FANCD2 | chr3:10088408-10088408 | NM_001018115 | c.1278+1(exon15)delG | splicing | rs750338758 |
| 3 | GOLGA8K | chr15:32685258 | NM_001282493 | c.1618G>A(exon17) | p.A540T | rs374107209 |
| 4 | KRTAP10-1 | chr21:45959556-45959557 | NM_198691 | c.477(exon1)_c.478(exon1)insCA | p.S160Hfs*? | na |
| 5 | KRTAP10-1 | chr21:45959558-45959559 | NM_198691 | c.475(exon1)_c.476(exon1)del | p.D159Ffs*9 | na |
| 6 | PCDHB8 | chr5:140558313-140558314 | NM_019120 | c.698(exon1)_c.699(exon1)insCC | p.V233Vfs*27 | na |
| 7 | ZDHHC11B | chr5:733915-733916 | NM_001351303 | c.974(exon12)_c.975(exon12)insTT | p.P325Pfs*9 | rs750289459 |
| 8 | C10orf129 | chr10:96961794-96961794 | NM_207321 | c.245(exon3)delA | p.E82Efs*10 | rs201108997 |
| 9 | KRT16 | chr17:39766663 | NM_005557 | c.1200C>A(exon6) | p.Y400X,74 | na |
| 10 | LOC732265 | chr15:74392546-74392546 | XM_003118667 | c.4(exon1)delT | p.Y2Mfs*6 | rs3214584 |
| 11 | RADIL | chr7:4875988 | NM_018059 | c.783+1G>A(exon3) | NA | na |
| 12 | ACE | chr17:61574284 | NM_001178057 | c.1784C>T(exon12) | p.T595M | rs12720742 |
| 13 | ADRA2A | chr10:112838217 | NM_000681 | c.463G>A(exon1) | p.E155K | rs373186564 |
| 14 | ATP6V0A2 | chr12:124221796 | NM_012463 | c.1016G>A(exon9) | p.R339H | rs74922060 |
| 15 | C10orf131 | chr10:97684601 | NM_001130446 | c.216G>A(exon6) | p.K72K | na |
| 16 | C4orf22 | chr4:81791190 | NM_001206997 | c.428T>C(exon5) | p.I143T | na |
| 17 | CARD14 | chr17:78172367 | NM_024110 | c.1828C>T(exon13) | p.R610C | rs371910172 |
| 18 | CRTAC1 | chr10:99661261 | NM_018058 | c.1132C>T(exon8) | p.R378C | rs200557763 |
| 19 | CYB5R1 | chr1:202932219 | NM_016243 | c.720G>T(exon8) | p.W240C | rs368686887 |
| 20 | DCHS1 | chr11:6647238 | NM_003737 | c.6644C>T(exon17) | p.P2215L | rs201703764 |
| 21 | DNAH11 | chr7:21751478 | NM_003777 | c.7005C>T(exon42) | p.S2335S | rs745645801 |
| 22 | GFI1B | chr9:135866403 | NM_004188 | c.959G>A(exon7) | p.R320Q | rs760858233 |
| 23 | IGSF22 | chr11:18728586 | NM_173588 | c.3455A>G(exon21) | p.N1152S | rs117051022 |
| 24 | KRTAP5-1 | chr11:1606121-1606150 | NM_001005922 | c.330(exon1)_c.359(exon1)del | p.G110_G120delinsG | rs775990209 |
| 25 | PDZD7 | chr10:102782123 | NM_001195263 | c.562C>T(exon5) | p.R188C | rs368583838 |
| 26 | PMM2 | chr16:8904949 | NM_000303 | c.361G>C(exon5) | p.E121Q | na |
| 27 | PMS2 | chr7:6022621 | NM_000535 | c.2008A>G(exon12) | p.K670E | na |
| 28 | RRP12 | chr10:99141549 | NM_015179 | c.1243C>T(exon11) | p.R415C | na |
| 29 | SLC26A9 | chr1:205898477 | NM_052934 | c.725T>C(exon7) | p.I242T | rs192547308 |
| 30 | SPATA20 | chr17:48631666 | NM_001258373 | c.1832G>A(exon15) | p.R611Q | rs199802020 |
| 31 | SZT2 | chr1:43908927 | NM_015284 | c.8317A>G(exon59) | p.M2773V | rs765172869 |
| 32 | TBC1D3B | chr17:34499750 | NM_001001417 | c.290G>T(exon6) | p.R97L | na |
| 33 | TIGD2 | chr4:90034397 | NM_145715 | c.272A>C(exon1) | p.K91T | rs757502186 |
| 34 | ZNF366 | chr5:71739995 | NM_152625 | c.1823G>T(exon5) | p.G608V | rs368802193 |
| 35 | ZNF717 | chr3:75786518 | NM_001128223 | c.2256T>G(exon5) | p.H752Q | rs113078821 |
| 36 | ZNF717 | chr3:75788245 | NM_001128223 | c.529G>A(exon5) | p.G177R | rs79835104 |
| 37 | OR2T29 | chr1:248722767 | NM_001004694 | c.26A>G(exon1) | p.N9S | rs764893066 |
| 38 | KRTAP2-1 | chr17:39203222 | NM_001123387 | c.298C>T(exon1) | p.Q100X,29 | rs370086433 |
| 39 | CTBP2 | chr10:126691628-126691628 | NM_022802 | c.1879(exon3)delC | p.L627Sfs*12 | na |
| 40 | RADIL | chr7:4875988 | NM_018059 | c.783+1G>A(exon3) | splicing | na |
