# Supplementary material for: The identification and characterization of the p.G91 deletion in CRYBA1 in a Chinese family with congenital cataracts
Source: BMC Med Genet. 2019 Sep 5;20:153. doi: 10.1186/s12881-019-0882-z (PMC6727356; doi:10.1186/s12881-019-0882-z)
Supplement: Supplementary file 2 — Cell Line Authentication Report STR Profiling. (PDF 221 kb) [file 12881_2019_882_MOESM2_ESM.pdf]

Cell Line Authentication Report
STR Profiling

Sample Type: Cell Line

Sample from: Eye & Ent Hospital Of Fudan University

Testing Method: STR Genotyping

Report Time: May16, 2017

COMPANY STATEMENT

1. THIS REPORT IS ONLY RESPONSIBLE FOR THE SAMPLES ANALYZED.
2. THE TESTING RESULTS AND THE ORGANIZATION NAME WILL NOT BE USED FOR ADVERTISEMENT, COMMERCIAL EXHIBITIONS, COMMERCIAL PERFORMANCE AND OTHER COMMERCIAL ACTIVITIES.
3. OBJECTIONS SHOULD BE RAISED WITHIN FIFTEEN DAYS AFTER THE RECEIPT OF THIS REPORT.
4. THE PAPER REPORT WITH CONTENT ALTERING, ADDING OR WITHOUT THE STAMPED SEAL OF THE COMPANY ARE INVALID.

Testing Company: Shanghai Biowing Applied Biotechnology Co. Ltd

Address: Room 4F, 8 th Buiding,Guiguo Garden,NO.471 Guiping Road,Caohejing Development Zone,Shanghai

Tel: +86-021-33559491

Contact: YiQun Chen

E-mail: biowing@vip.163.com

Cell Line Authentication – STR Profiling Report

Sample code

Table 1. Sample Code

Customer's code	Company Code
B3	20170508

Sample Number:1

Sample Type: Cell line

Testing Type: STR

Sample From: Eye & Ent Hospital Of Fudan University

Testing Method:

DNA was extracted by a commercial kit from CORNING (AP-EMN-BL-GDNA-250G). The twenty STRs including Amelogenin locus were amplified by six multiplex PCR and separated on ABI 3730XL Genetic Analyzer. The signals were then analyzed by the software GeneMapper.

Data Interpretation:

Cell lines were authenticated using Short Tandem Repeat (STR) analysis as described in 2012 in ANSI Standard (ASN-0002) by the ATCC Standards Development Organization (SDO) and in Capes-Davis et al., Match criteria for human cell line authentication: Where do we draw the line? Int J Cancer.2013;132(11):2510-9.

Test Results:

1. Result

Table 2. Matching information on the cell lines

Sample Code	Multi-allele	Cell line matched	Cell Bank	EV	Percentage
20170508	Yes	SRA 01/04	DSMZ	1	9/9

- **Multi-allele means some STR contain more than two loci.**

2. Sample Description

20170508:

A. The STR results showed that multiple alleles were found in this cell line, and no cross contamination of human cells was found in the cell line.

B. The DNA of the cell lines found to perfect match the type of cell lines in a cell line retrieval, DSMZ database shows that cells called **SRA 01/04,** corresponding to the cell number **RCB1591.**

Cell No.	Cell name	Locus names								
		D5S818	D13S317	D7S820	D16S539	VWA	TH01	AM	TPOX	CSF1PO
Query (Your Cell)		*11, 12, 13*	*10, 10*	*10, 11, 13*	*10, 10*	*17, 20*	*6, 7*	*x, y*	*8, 8*	*11, 13, 15*
RCB1591	SRA 01/04	11, 12, 13	10, 10	10, 11, 13	10, 10	17, 20	6, 7	X, Y	8, 8	11, 13, 15

3. Genotyping Result

Table 3. STR and Amelogenin Genotyping Results of Cell line 20170508

Marker	Sample				Cell Bank information		
	Allele1	Allele2	Allele3	Allele4	Allele1	Allele2	Allele3
D5S818	11	12	13		11	12	13
D13S317	10	10			10	10	
D7S820	10	11	13		10	11	13
D16S539	10	10			10	10	
VWA	17	20			17	20	
TH01	6	7			6	7	
AMEL	X	Y			X	Y	
TPOX	8	8			8	8	
CSF1PO	11	13	15		11	13	15
D12S391	19	22					
FGA	20	22.2					
D2S1338	23	24					
D21S11	27	28					
D18S51	13	13					
D8S1179	14	15					
D3S1358	14	15					
D6S1043	16	18					
PENTAE	7	21					
D19S433	13	14					
PENTAD	9	12	13				

Others:

1. Genotyping Strategy and Site Distribution

Attached Table. Experimental Strategy and Sites

	**Strategy 1**	**Strategy 2**	**Strategy 3**	**Strategy 4**	**Strategy 5**	**Strategy 6**
1	TH01	AMEL	TPOX	D3S1358	PENTAE	D19S433
2	D12S391	D5S818	VWA	D13S317		PENTAD
3	D7S820	D2S1338	D8S1179	D6S1043		
4	CSF1PO	D21S11		D16S539		
5	FGA	D18S51				

The allele match algorithm compares the 8 core loci plus amelogenin only, even though alleles from all loci will be reported when available.

2. DSMZ tools was used to carry on the cell line comparison, which contains 2455 cell lines STR data from ATCC, DSMZ, JCRB ,ECACC，GNE and RIKEN databases. If the cell is not included in the above cell library, users need to compared with other databases.

Technician:Menglu Shen
Check: Yang Bai
Person in Charge: Yiqun Chen

Issue date: May16, 2017, 2017
