# Supplementary figures and images for: The identification and characterization of the p.G91 deletion in CRYBA1 in a Chinese family with congenital cataracts
Source: BMC Med Genet. 2019 Sep 5;20:153. doi: 10.1186/s12881-019-0882-z (PMC6727356; doi:10.1186/s12881-019-0882-z)

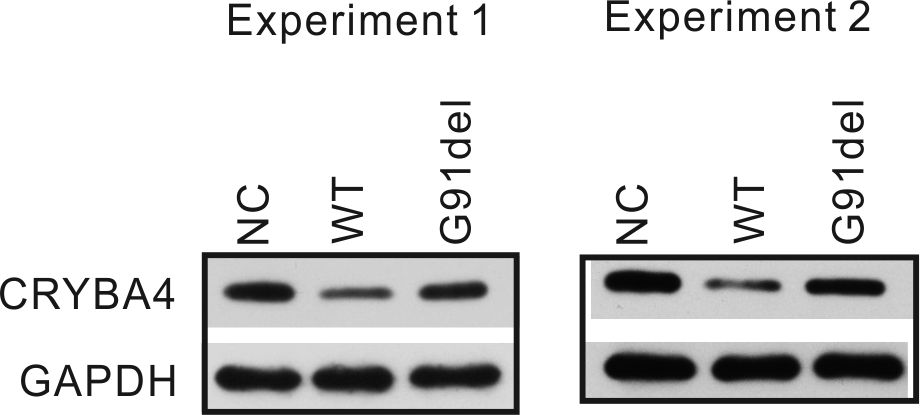

Supplement: Supplementary file 3 — Figure S1. Overexpression of CRYBA1 would reduce CRYBA4 production. After the WT and deleted forms of CRYBA1 cDNA constructs were transfected into SRA cell lines, the protein levels of CRYBA4 were measured by Western blot. GAPDH was used as internal control. NC, negative control: transfection reagent only. (JPG 42 kb) [file 12881_2019_882_MOESM3_ESM.jpg]

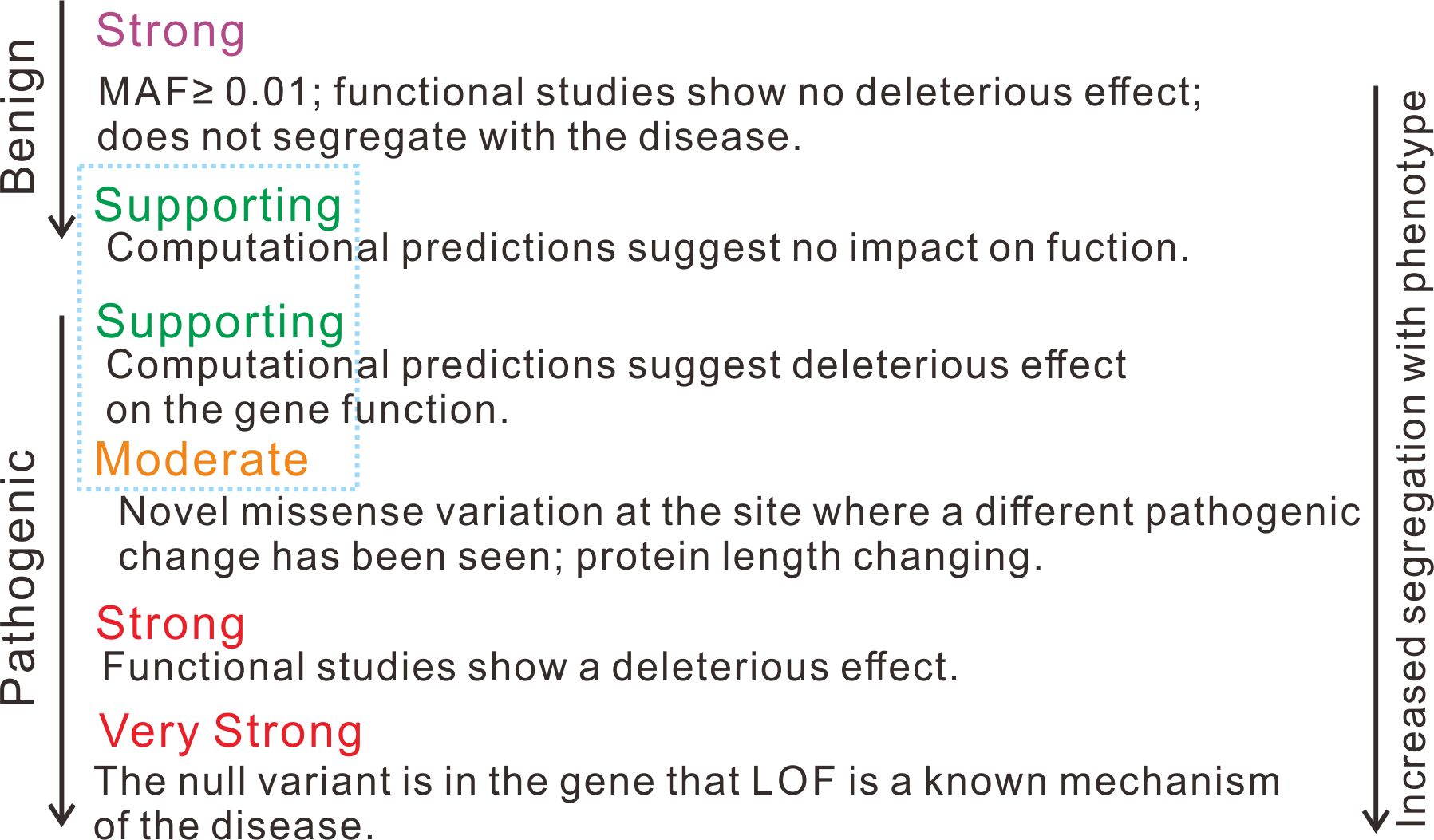

Supplement: Supplementary file 4 — Figure S2. Six categories with increasing severity of pathogenicity. Re-edited from Fig. 1 in [16]. (JPG 226 kb) [file 12881_2019_882_MOESM4_ESM.jpg]
